# A low α-linolenic intake during early life increases adiposity in the adult guinea pig

**DOI:** 10.1186/1743-7075-7-8

**Published:** 2010-01-29

**Authors:** Etienne Pouteau, Olivier Aprikian, Catherine Grenot, Denis Reynaud, Cecil Pace-Asciak, Claude Yves Cuilleron, Eurídice Castañeda-Gutiérrez, Julie Moulin, Gregory Pescia, Carine Beysen, Scott Turner, Katherine Macé

**Affiliations:** 1 Nestlé Research Centre, PO Box 44, Vers-Chez-Les-Blanc, 1000 Lausanne 26, Switzerland; 2 Institut National de la Santé et de la Recherche Médicale, ERM 322, Hôpital Debrousse, 69322 Lyon, France; 3 Research Institute, Rm 2032F, E McMaster Bldg, The Hospital for Sick Children, Toronto, Canada; 4 KineMed Ltd, 5980 Horton Street, Suite 400, Emeryville, CA 94608, USA

## Abstract

**Background:**

The composition of dietary fatty acids (FA) during early life may impact adult adipose tissue (AT) development. We investigated the effects of α-linolenic acid (ALA) intake during the suckling/weaning period on AT development and metabolic markers in the guinea pig (GP).

**Methods:**

Newborn GP were fed a 27%-fat diet (w/w %) with high (10%-ALA group), moderate (2.4%-ALA group) or low (0.8%-ALA group) ALA content (w/w % as total FA) until they were 21 days old (d21). Then all animals were switched to a 15%-fat diet containing 2% ALA (as total FA) until 136 days of age (d136).

**Results:**

ALA and docosapentaenoic acid measured in plasma triglycerides (TG) at d21 decreased with decreasing ALA intake. Total body fat mass was not different between groups at d21. Adipose tissue TG synthesis rates and proliferation rate of total adipose cells, as assessed by ^2^H_2_O labelling, were unchanged between groups at d21, while hepatic de novo lipogenesis was significantly 2-fold increased in the 0.8%-ALA group. In older GP, the 0.8%-ALA group showed a significant 15-%-increased total fat mass (d79 and d107, p < 0.01) and epididymal AT weight (d136) and tended to show higher insulinemia compared to the 10%-ALA group. In addition, proliferation rate of cells in the subcutaneous AT was higher in the 0.8%-ALA (15.2 ± 1.3% new cells/5d) than in the 10%-ALA group (8.6 ± 1.7% new cells/5d, p = 0.021) at d136. AT eicosanoid profiles were not associated with the increase of AT cell proliferation.

**Conclusion:**

A low ALA intake during early postnatal life promotes an increased adiposity in the adult GP.

## Introduction

Worldwide, about 1.3 billion of individuals suffer of excessive adiposity, 940 million are overweight and 400 million are obese [1]. This epidemic is driven by a widespread energy imbalance, with energy intake exceeding energy expenditure. Nevertheless, individual differences in the susceptibility to gain an excess of fat mass, in obesogenic environments exist. In addition to genetic factors there is a growing body of evidence, from epidemiological and animal studies, that perinatal nutrition may substantially predispose to the development of obesity. Fetal over and under-nutrition promotes adiposity later in life in different animal models [2,3] and infants small for gestational age appear to have a higher risk of developing adult obesity [4]. Our understanding of the factors and mechanisms, by which perinatal nutrition impacts later susceptibility to obesity, remains limited. Nevertheless, the early nutritional environment may set adipose tissue growth and function towards either fat storage or oxidation, later in life [5]. There is some evidence that the composition of dietary fatty acids and especially the essential polyunsaturated fatty acids (PUFA), linoleic acid (LA; C18:2n-6) and α-linolenic acid (ALA; C18:3n-3) and their respective long-chain products, arachidonic acid (AA; C20:4n-6), eicosapentaenoic acid (EPA; C20:5n-3) and docosahexaenoic acid (DHA; C22:6n-3) influence early adipose tissue development. These effects may be mediated by eicosanoids, and more especially by prostaglandins (PG; PGD_2_, PGE_2_, PGF_2α_, PGI_2_), involved in adipose tissue growth and metabolism and dependent on the long-chain PUFA availability [6]. Animal studies showed that the intake of a high LA/ALA ratio during fetal and postnatal life promotes early adipose tissue growth and obesity [7,8]. Nevertheless, since enhancing the LA/ALA ratio in the diet implies either a substantial increase of LA [8], a decrease of ALA [7] or both [8], it is difficult to determine if the n-6 PUFA promote adipose tissue development or if the n-3 PUFA have anti-adipogenic properties. Because, the adipogenic effects of the n-6 PUFA remain controversial [9], we hypothesized that a low level of ALA rather than an excess of LA leads to an increased adiposity. In order to test part of this hypothesis, 3 groups of newborn guinea pigs (GP) were fed until 21 days of age with milk and pellets containing either a high (10% total FA), medium (2.4% total FA) or low but adequate (0.8% total FA) ALA content corresponding respectively to a LA/ALA ratio of 2:1, 10:1 and 30:1. The short and long term consequences of such early postnatal dietary interventions on adipose tissue development and metabolic markers were determined.

## Materials and methods

### Animals and diets

All experimental procedures were reviewed and approved by the Swiss authorities ethical committee (authorization 1910, "Service vetérinaire du canton de Vaud"). Male and female Dunkin-Hartley guinea pigs (450-500 g of body weight) were purchased from Charles-River (Lyon, France) and housed together for mating. The guinea pigs received a commercial chow diet (Kliba 3420, Promivi Kliba SA, Kaiseraugst, Switzerland) during mating and gestation period. At birth, pups remained with their dams for 2 days, and afterwards the male pups were nourished with one of the following 27%-fat diets (w/w) differing in ALA content: 10%-ALA (high), 2.4%-ALA (moderate) or 0.8%-ALA (low, w/w % as total FA) until d21. From d2 to d4 the pups received reconstituted milk formula and from d5 to d21 food pellets. Milk formula and pellet composition is provided in Table S1, Additional file 1 and the fatty acid profile is detailed in Table S2, Additional file 2. From d21 to d136, all guinea pigs received a 15%-fat diet based on D22451 formulation (Research Diets, New Brunswick, NJ). Coconut oil, corn oil, palm oil and rapeseed oil (Sofinol S.A. Manno, Switzerland) were mixed with ingredients of D224521 diet. Corn starch was added (Schweizerhall Chemie, Basel, Switzerland) and lard, soybean oil and maltodextrin were removed. The diet contained 32.6% saturated fat, 39.2% monounsaturated fat and 28.2% PUFA among them 26% LA and 2% ALA. Twenty grams of timothy hay (Bio-Serv, Frenchtown, NJ) was added weekly.

### Study design

The male pups were randomized by body weight and assigned to 10%-ALA group (n = 25), 2.4%-ALA group (n = 20) or 0.8%-ALA group (n = 25) during the suckling/weaning period until d21, and then all groups were fed a common diet until the end of the experiment. Body weight and food intake were recorded every morning until d21 and then every 3 d until d136. Guinea pigs were euthanized by exsanguination under isofluorane anesthesia at d21 (10 pups for 10%-ALA and 0.8%-ALA groups and 5 pups for 2.4%-ALA group) and at d136 (15 animals/group). Ten animals from 10%-ALA and 0.8%-ALA groups received deuterated water (^2^H_2_O) for determination of cell proliferation and lipid synthesis rate at d21 and d136 [10-12]. These animals received ^2^H_2_O (99%, sterilized 0.9% NaCl, 35 mg/g of BW) intraperitoneally 5 d before euthanasia, thereafter, they drank 8% deuterated water ad libitum until euthanasia. Blood (200 μL) was collected from the aorta in heparinised vials. Bone marrow from the femur of the hind limb (flushed by 0.9%-NaCl-water using a 25G-needle-seringe), adipose tissue, and liver were collected (about 50 mg) and weighed. All tissues and plasma were kept at -80°C until analyses. Additionally and for the other animals, venous blood was collected from the saphenous vein prior sacrifice for metabolite analyses. Retroperitoneal, epididymal, interscapular and subcutaneous adipose tissue, as well as the liver and the pancreas were collected and weighed. The body composition of animals was determined at regular intervals.

### Analytical procedure

#### Fatty acid (FA) analysis

Total lipids of diet and plasma were extracted, and FA profile of plasma phospholipids (PL) and TG was determined in 4 animals per group as previously described [9].

#### Biochemical assays

Plasma TG, FFA and glucose concentrations were analyzed with commercially available kits (Roche Diagnostic, Basel, Switzerland; Wako Chemicals, Richmond, VA and Sigma, Buchs, Switzerland, respectively).

#### Guinea pig insulin purification and immunization

Guinea pig insulin was isolated from frozen pancreas according to Yip & Ottensmeyer [13]. Purification was improved by three successive chromatographies during which the insulin-containing fractions were detected by measurement of the radioactivity of 125I bovine insulin added as tracer. The overall yield was 2.5 mg of purified insulin per 20 pancreas of guinea pig. Degree of purity of purified insulin was first controlled by reverse-phase HPLC (300-5-C18 column, Macherey-Nagel, France) that showed the presence of a major protein peak (>90%) and by SDS-PAGE using the Schägger and von Jagow method [14], adapted for the separation of low molecular mass proteins and peptides (≤ 10 kDa). After silver nitrate staining, the gel showed the presence of a single intense band with an apparent mass of 6 kDa, in agreement with the molecular mass of insulin. The degree of purity was higher than 90%. An electrospray mass spectrum of the purified fraction showed the presence of three multicharged [M+nH+] ions corresponding to a single protein of molecular mass M of 5778.5, in agreement with the mass of 5778.4, calculated for guinea pig insulin. A radioactive insulin tracer was prepared by radioiodination of 3 μg of purified insulin with 400 μCi of 125I iodine using the chloramine T method in the conditions employed for the radiolabeling of bovine insulin. Radiolabeled insulin was purified on Sephadex G50 column and a fraction corresponding to the top of the radioactive peak was collected and used as tracer to test rabbit antibodies. Immunization of rabbits was initiated by 4 successive injections. After the first six months of immunization titers of both rabbits were measured at 1/10,000 and remained stable up to now (one year of immunization). An estimation of the specificity was done by the determination of cross-reactivity with human, bovine, rabbit and mouse sera that showed no cross-reaction with human, bovine and mouse sera.

#### Radio-immunoassay (RIA) for guinea pig insulin

RIA was developed with rabbit antiserum incubated with the radioactive 125I insulin tracer and different concentrations of purified insulin as standard hormone. The assay was performed with serum or plasma obtained after collection of blood samples on EDTA. Owing to the low concentrations observed in young guinea pigs, the standard curve was established with eight concentrations of standard insulin ranging from 0.5 to 100 ng.mL-1. The best standard curve was obtained with an amount of 35'000-cpm/per tubes. Rabbit antiserum at a dilution of 1/10,000, were found to be suitable for the establishment of the standard curve. Incubation for 20 hours at 20°C led to the highest bound fractions along with the lowest non-specific binding. The RIA program of the gamma counter was employed using the LOGIT/log curve fitting. Reproducibility and accuracy (spiking and dilution test) controls were performed to assess of the values of insulin concentration. The RIA developed with rabbit antibodies showed a sensitivity of 1 ng.mL^-1^.

#### The pancreatic insulin content

The insulin was extracted from pancreas by acid-ethanol solution at -20°C overnight [15]. The insulin content in the supernatant was assessed by above developed RIA method and expressed in ng.μg^-1 ^of protein.

#### Eicosanoid analysis

Adipose tissue (50 mg) was homogenized with distilled ethanol (3 mL) as previously detailed [9]. The lipid supernatant was then dried under nitrogen, dissolved with 50 μL of ethanol/1 mL water and extracted with ethyl acetate after acidification. The ethyl acetate extract was dried and dissolved in acetonitrile/water for analysis of eicosanoids by liquid chromatography-mass spectrometry (LC-MS/MS) using selected ion monitoring [9].

#### Determination of deuterium enrichments in DNA and lipids

The measure of ^2^H-enrichments in DNA and in lipids has been previously detailed [11,16,17]. Briefly, cellular DNA from the adipose depot (> 20 mg of tissue) was isolated. After sequential hydrolysis genomic deoxyribose moiety was obtained from deoxyadenosine. Deoxyribose was then acetylated into pentose-tetraacetate derivative and analyzed by gas chromatograph/mass spectrometer (GC/MS, Hewlett-Packard, Palo Alto, CA) for measurement of ^2^H-isotope enrichments [17]. Separately, adipose TG were isolated for analyses of ^2^H-enrichments of TG-glycerol and of TG-palmitate by GC/MS as described elsewhere [12,18-20]. Deuterium enrichment in body water was measured from plasma (20 μL) as described previously [21]. Standard curves of known ^2^H-enrichments (EM1 in % excess) of the metabolites were used to assess ^2^H-isotope enrichments [20].

#### Calculations of fractional synthesis rates

Use of ^2^H_2_O incorporation for the measurements of DNA replication rate (cell proliferation rate), TG-glycerol synthesis rate (all-source TG turnover) and TG-palmitate synthesis rate (all-source DNL) has been described [12,20]. The method counts the cell divisions that occurred during the labeling period. The fractional proliferation rate of adipose tissue cells (fractional synthesis rate, FSR_cell _in % new cell/5 d) identical to the fraction of newly synthesized DNA was calculated as the ^2^H-enrichment in adipose tissue DNA divided by ^2^H-enrichment in bone marrow DNA [11,22]. The principle for calculating the fractional synthesis rate of TG (FSR_TG_) in adipose tissues is that TG that were synthesized from endogenous alpha-glycerol phosphate before ^2^H_2_O was present, do not contain covalent C-^2^H label in their glycerol moiety, thereby allowing the proportion of newly synthesized vs. pre-existing TG to be assessed [20]. Glycerol kinase in the liver overestimates the calculation of FSR_TG _in the liver. The FSR_TG _(in % new TG/5 d) was calculated from the ratio of EM1_TG _to , where EM1_TG _is ^2^H-enrichment for glycerol at 5 d, and  is the maximal ^2^H-enrichment for glycerol [12]. The fractional synthesis rate of palmitate (DNL contribution to palmitate, FSR_palmitate _in % new palmitate/5 d) in TG was calculated from the label ^2^H-incorporation from water into TG-palmitate using MIDA (mass isotopomer distribution analysis) [23].

#### Body composition

Body composition of pups was determined at 2, 21, 51, 79, 107 and 128 days of age using a 0.05-Tesla-Magnetic Resonance Imaging (EchoMRITM 400, Echo Medical Systems, Houston, TX, US). Calculation and interpretation were performed according to Taicher GZ [24] and Tinsley FC [25].

### Statistical analysis

According to distribution of data, non-parameteric tests were used for most outcomes (Global kruskal-Wallis tests and Mann-Whitney-Wilcoxon tests for pairwise comparisons). Outcomes were expressed as Median and Standard Error of the Median (SEmedian) based on the robust Sn of Rousseeuw. Comparisons of tissues based on eicosanoids were done using Wilcoxon Signed Rank tests. A low number of data on the FA profile was sufficient to illustrate differences, although low number of data involves cautious interpretation. A parametric mixed model was used for NMR analysis, with subject as random effect. These parameters are thus described by the Mean and the Standard Error of the Mean (SEM).

## Results

Food intake was not different between groups. The mean cumulative food intake was 118.1 ± 5.8 g (10%-ALA), 124.6 ± 7.0 g (2.4%-ALA) and 125.1 ± 10.7 g (0.8%-ALA group) from d9 to d18 and was 3013.3 ± 228.7 g (10%-ALA), 3138.6 ± 166.2 g (2.4%-ALA) and 3231.4 ± 119.9 g (0.8%-ALA group) from d23 to d129.

**Figure 1:**
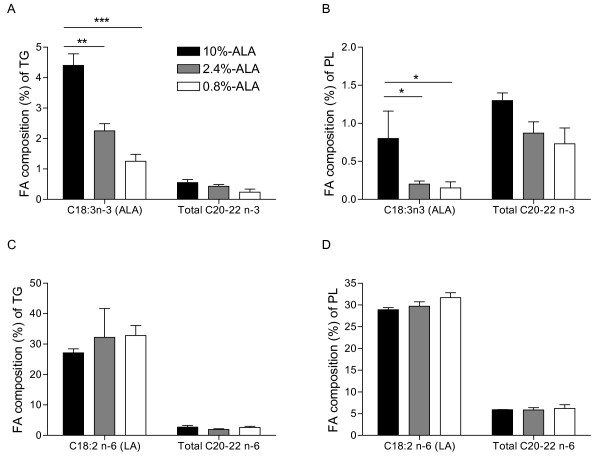
**Fatty acid composition (mol %) of plasma triglycerides (TG) and phospholipids (PL) at d21**. Proportion of ALA and total other long-chain n-3 PUFAs (C20:3n-3, C20:5n-3, C22:5n-3, C22:6n-3) in plasma TG **(A) **and PL **(B)**. Proportion of LA and total other long-chain n-6 PUFAs (C20:2n-6, C20:3n-6, C20:4n-6, C22:2n-6, C22:4n-6) in plasma TG **(C) **and PL **(D)**. Data are medians ± SEmedian, n = 4-5 samples/group in duplicate. LA, linoleic acid; ALA, alpha-linolenic acid. * p < 0.05, ** p < 0.01, *** p < 0.001 between groups.

Fatty acid profile of plasma lipids was determined at the end of the dietary intervention period (d21). Inclusion of 10% ALA in the diet resulted in its increase in plasma TG and PL (TG: 4.40 ± 0.35%; PL: 0.80 ± 0.26%, figures 1A and 1B), but the increase was not significant with the addition of 2.4% ALA (TG: 2.25 ± 0.23%; PL: 0.20 ± 0.01% and TG: 1.25 ± 0.37%; PL: 0.15 ± 0.07% for 2.4% ALA and 0.8% ALA, respectively). The proportion of total C20-22 n-3 PUFA, including eicosapentaenoic acid (EPA), docosapentaenoic acid (DPA) and docosahexaenoic acid (DHA), in plasma TG (figure 1A) and PL (figure 1B) tended to decrease with decreasing ALA intake, but this effect did not reach statistical significance (p = 0.09 and p = 0.07 for PL and TG, respectively), except for DPA in TG and for EPA in PL. The 0.8%-ALA group (0.01 ± 0.01 mol %) had lower DPA in TG than both other groups (2.4%-ALA: 0.20 ± 0.12, p < 0.05 and 10%-ALA: 0.20 ± 0.01 mol %, p = 0.06). For EPA in PL, there was a significant difference between 10%-ALA group (0.10 ± 0.01 mol %) and 0.8%-ALA group (0.01 ± 0.01 mol %, p = 0.018). As expected, there was no difference in plasma LA between groups and increasing ALA intake did not modify the proportion of n-6 PUFA (figures 1C and 1D).

The body weight (BW; 10%-ALA: 869.3 ± 54.2 g; 2.4%-ALA: 860.7 ± 29.6 g and 0.8%-ALA group: 902.3 ± 25.7 g at d136) and BW gain (data not shown) did not differ between groups during the course of the experiment.

**Figure 2:**
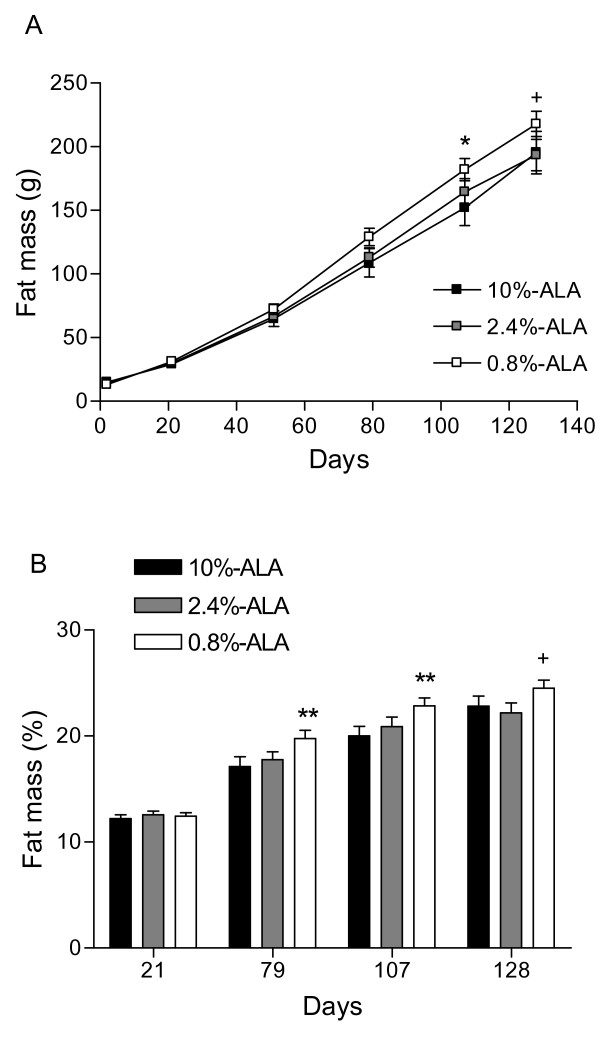
**Fat mass evolution**. **(A) **Fat mass (g) evolution from d21 to 128. **(B) **Fat mass (% of body weight) at 21, 79, 107 and 128 days. Data are means ± SE, n = 15-20 guinea pigs/group. ^+ ^indicates p < 0.05 between the 0.8%-ALA and 2.4%-ALA or 10%-ALA groups. * indicates p < 0.05 and ** indicates p < 0.01 between the 0.8%-ALA and 10%-ALA groups.

Decreased intake of ALA during early life resulted in increased body fat at 136 days; Figure 2 shows the fat mass evolution during the course of the study expressed as g (figure 2A) or % body weight (figure 2B). While no differences were observed at the end of the dietary intervention period (d21), older guinea pigs in the 0.8%-ALA group had or tended to have more fat mass than those in the 10%-ALA group at d79 (figure 2A: +20.3 g, p = 0.08; figure 2B: +2.6%, p < 0.01), at d107 (+29.9 g, p < 0.05; +2.8%, p < 0.01) and at d128 (+22.6 g, p = 0.05; +1.7%, p = 0.08) and than those in the 2.4%-ALA group at d128 (+24.2 g, p < 0.05; +2.2%, p < 0.05).

Likewise, at d 136, epididymal AT was or tended to be heavier in the 0.8%-ALA than in the 10%-ALA (+0.19 g/100 g BW, p < 0.05) and the 2.4%-ALA group (+0.16 g/100 g BW, p = 0.05). The interscapular AT weight also was higher in the 0.8% than in the 2.4%-ALA groups (+0.18 g/100 g BW, p < 0.01). The weights of epididymal, interscapular and retroperitoneal AT relative to BW were not different between groups at d21 (Table S3, Additional file 3). The liver weight as well as the pancreas weight did not differ between groups at d21 and d136 (data not shown).

The fractional proliferation rates (FSR) of cells, measured in the retroperitoneal and epididymal AT, were unchanged between 10%-ALA and 0.8%-ALA groups at both d21 and d136 (Table 4, Additional file 4). Nonetheless, the cell proliferation rate in the subcutaneous AT was higher in the 0.8%-ALA group compared to the 10%-ALA group at d21 (2.1-fold higher; not significant) and d136 (1.8-fold higher; p = 0.021) (Table 4, Additional file 4).

The de novo lipogenesis (DNL) and synthesis rate of TG in the different adipose fat pads did not differ between the 10%- and 0.8%-ALA groups at d21 (Table 5, Additional file 5). A higher hepatic DNL (P < 0.001) was observed at d21 in guinea pigs fed 0.8%-ALA intake compared to 10%-ALA intake (Table 5, Additional file 5). No difference was observed in DNL or synthesis rate of TG in hepatic or adipose tissues between groups at d136 (Table 5, Additional file 5).

The arachidonic acid (AA) and most eicosanoid concentrations determined in retroperitoneal and subcutaneous fat pads were not different between the groups (Table 6, Additional file 6). An exception was observed for PGE2 and PGJ2 that were both significantly higher after medium vs low ALA intake at d136 (Table 6, Additional file 6). In order to evaluate the AT differences, the groups were combined and the eicosanoid profile was compared between the fat depots at both ages. At d21, AA, 6-keto-PGF_1α _and PGJ_2 _concentrations were 1.6-fold (244.7 ± 24.9 vs 149.6 ± 14.0 ng/50 mg AT, p < 0.01), 3.7-fold (1.95 ± 0.10 vs 0.52 ± 0.09 ng/50 mg AT, p < 0.0001) and significantly (0.015 ± 0.001 vs 0.001 ± 0.001 ng/50 mg AT, p < 0.0001) higher in the retroperitoneal AT when compared to the subcutaneous one, respectively. At d136, the difference of PGJ_2 _concentrations was even higher between the retroperitoneal (1.53 ± 0.35 ng/50 mg AT) and subcutaneous (0.01 ± 0.01 ng/50 mg AT, p < 0.0001) AT. In addition, 6-keto-PGF_1α_, PGE_2 _and PGF_2α _were 1.5-fold (p < 0.001), 23-fold (p < 0.0001) and 1.6-fold (p < 0.0001) higher in the retroperitoneal vs subcutaneous AT (data not shown).

**Figure 3:**
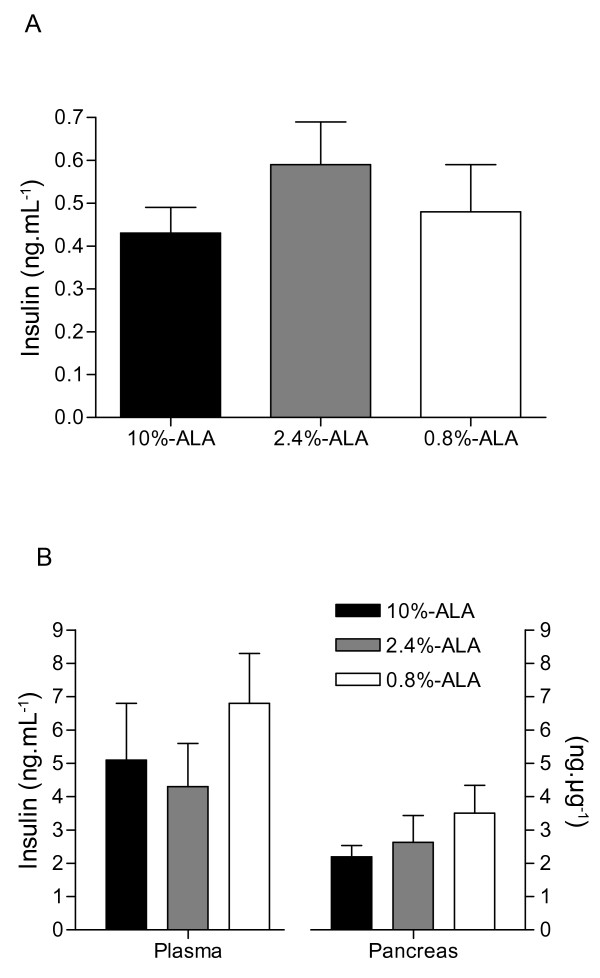
**Plasma insulin concentrations and pancreatic insulin contents**. **(A) **Plasma insulin at d21, n = 5 guinea pigs/group. **(B) **Plasma and pancreas insulin at d136, n = 10-15 guinea pigs/group. Data are medians ± SEmedian, no significant difference.

The plasma FFA were not different between groups at d21 (data not shown) or at d136 (10%-ALA, 17.6 ± 0.7; 2.4%-ALA, 18.0 ± 0.6 and 0.8%-ALA group, 18.0 ± 1.1 mg.dL^-1^). The plasma TG concentrations were unchanged at d21 (data not shown) but were 1.3-fold lower in the 0.8%-ALA vs 10%-ALA (1.31 ± 0.05 vs 1.78 ± 0.21 mmol.L^-1^, p < 0.05) and vs 2.4%-ALA group (1.61 ± 0.15 mmol.L^-1^, not significant), at d136. There were no differences in plasma insulin concentrations between groups at the end of the suckling/weaning period (figure 3A). At d136 (figure 3B) the 0.8%-ALA group (6.8 ± 1.5 ng.mL^-1^) showed about 1.5-fold higher concentrations of plasma insulin when compared with other groups (2.4%-ALA, 4.3 ± 1.3 and 10%-ALA group, 5.1 ± 1.7 ng.mL^-1^, not significant). Similar results were observed regarding the pancreatic insulin content. The 0.8%-ALA group showed about 1.6-fold increase in the pancreatic insulin content (3.5 ± 0.8 ng.μg^-1 ^of protein) when compared to the 10%-ALA group (2.2 ± 0.3 ng.μg^-1 ^of protein, p = 0.06, not significant) at d136 (figure 3B). There were no significant differences in plasma glucose between groups at d21 (data not shown) and neither at d136 (10%-ALA, 112.0 ± 3.3; 2.4%-ALA, 110.0 ± 6.7 and 0.8%-ALA group, 104.5 ± 4.6 mg.dL^-1^).

## Discussion

To our knowledge this is the first study showing that dietary ALA intake during the suckling/weaning period influences fat mass development later in life; the lowest dietary ALA intake programming the highest adiposity in the adult guinea pig.

In our study, the adipose tissue development was not impacted during the period of dietary intervention with different ALA levels. Indeed, the fat mass and adipose tissue weight at d21 were not different between the high, moderate and low ALA fed guinea pigs. Our results are in agreement with Korotkova *et al*. who showed that offspring of rat dams, fed a 6.2% ALA (total FA) enriched diet during the gestation and suckling period, did not differ in AT weight at 3 weeks of age compared to a low ALA diet with the same amount of dietary lipids [8]. On the contrary, the dietary intake of chia seed (rich in ALA) decreased adiposity in adult rats fed an obesegenic diet [26]. Interestingly this effect was associated with a large increase in plasma lipid n-3 LC-PUFAs (EPA and DHA), known to have anti-adipogenic effects [27], probably through inhibition of the proliferation of adipose tissue cells [27]. While no one can exclude that ALA has its own specific physiological effects on AT development, it is tempting to speculate that these effects are more dependent on its long-chain products. Unlike other rodents and similarly to humans, the efficiency of ALA conversion into its long-chain products is limited in guinea pigs [28]. As previously shown in AT and other tissues of the guinea pigs [29], we observed in our study a higher ALA proportion in plasma TG and phospholipids with an increasing ALA intake but with a limited increase of >C20 n-3 PUFA. Indeed, EPA, DPA or DHA represents less than 0.8% of total FA in our study, even in the group with the highest ALA intake, and could explain the absence of impact on the adipose tissue development of the 21-day old guinea pigs. Previous studies have indicated that n-3 LC-PUFAs enriched diets diminish the hepatic enzymatic activities of endogenous fatty acids synthesis [30] associated with a decrease in plasma TG [31,32]. In the present study, the high ALA intake induced a 2-fold reduction of fractional DNL in the liver at d21 with no impact on total plasma TG concentration. A higher >20 n-3 PUFA plasma concentration during the high ALA intake could modulate transcription factors in the liver. It is possible that that >20 n-3 PUFAs reduce DNL by down-regulating SREBP-1 (sterol regulatory element-binding protein-1) and activating PPAR alpha (peroxisome proliferator-activated receptor alpha) as shown by previous studies [33]. To explain no difference on total plasma TG, we considered that fatty acids for TG synthesis come from the DNL, the dietary source or from the adipose tissue lipolysis. Based on the calculation of the FA contribution from DNL to TG synthesis (ratio of DNL to TG FSR), we estimated that the contributions from the dietary source plus the adipose tissues were 72% and 87% for the low and high ALA groups, respectively [10]. Since dietary FA contributed equally between groups, plasma TG containing FA from the adipose tissue may have been greater in the high ALA group compared with low ALA group at d21. It is possible that more plasma TG containing FA from the adipose tissue was utilized by the liver or muscle (e.g. for oxidation). Further analysis of VLDL-TG (very large density lipoprotein-TG) secretion and removal rates in and from the circulation could clarify the unchanged plasma TG. Ultimately the fat mass was unchanged between the groups at the end of the suckling/weaning diet treatment.

The long-term adipogenic effect of a low ALA intake during early postnatal life seems to be more pronounced on subcutaneous than visceral AT. Indeed, the 2 to 3% differences of total fat mass found between the low and high ALA group at d107 and d128 may not be explained by the observed difference of epididymal fat pad weights, knowing that the visceral fat depots (calculated by summing the weight of epididymal, retroperitoneal and mesenteric AT) represent only about 18% of total fat mass in the adult GP. The subcutaneous adipose tissue which represents the major fat depot in adult (about 80%) revealed a significant increase in cell proliferation in adults after the 0.8% ALA versus 10% ALA postnatal diet treatment. No hyperplasia was noticed in other fat depots after 0.8% ALA intake. This supports a specific role of the subcutaneous adipose tissue. Previous studies indicated a direct effect of n-3 fatty acid enriched diet on decreasing adipocyte differentiation and increasing apoptosis in vitro [34] and in high-fat fed rats [35]. In the present study, we could not measure the deuterium enrichment decay in DNA to determine the apoptosis rate that could have been lowered in subcutaneous AT of the adult GP of low ALA group. On the contrary, we observed a clear imprinted effect of low ALA early intake on increasing the cell proliferation rate by 75% versus high ALA early intake in the subcutaneous AT of adult GP. In addition, we showed a transient increase of hepatic DNL in the low ALA group at d21 that could be involved in the imprinting mechanism. Further investigations are needed to clarify the mechanism.

Interestingly, the 0.8% ALA group had higher insulin at the end of the experiment, though the differences between groups did not reach statistical significance due to a high variability. This tendency for higher insulinemia is probably linked to an increased adiposity, as known for other animal species and human. We found a positive correlation between insulin (expressed as log) and fat mass in the adult guinea pigs (Pearson r = 0.77, p < 0.0001). Although all in a normal range [36], plasma TG were significantly higher in the high versus medium and low ALA groups at d136. This was not associated with an increased adipose tissue FSR of total TG in the high ALA group. Previous studies showed the capabilities of n-3 LC-PUFAs to modulate lipolysis and consequently alter TG level [27,37]. Although we described a possible imprinted effect in the present study, the increased plasma TG could be due to an increased lipolysis to support a lower fat mass in the adult GP of high ALA group.

The mechanism by which a low ALA intake programs an excess of adipose tissue is unclear. It may imply cell proliferation as indicated in the present study by the increase of fractional proliferation rate of cells in the subcutaneous AT. Because eicosanoids are modulated by LC-PUFAs and are involved in the differentiation and proliferation of adipose tissue cells we expected to see differences between groups [38]. Beside the large differences between the types of fat depots (visceral vs subcutaneous) on eicosanoid levels, we found only minor changes between groups and none in subcutaneous AT. Further studies are definitely needed to understand the mechanism by which early dietary ALA impacts adipogenesis and lipogenesis later in life. The Guinea Pig represents a good model to the human term newborn [39] but shows some experimental limitations due to the lack of commercially available immunological kits and epigenetic markers. This may change with the recent first release of the genome sequencing and assembly of the Guinea Pig [40]. Future investigations using the Guinea Pig model will help understanding the mechanisms by which early nutrition may program adiposity later in life. It would be also interesting to evaluate whether the programming effect of a low ALA intake may have been more pronounced with dietary interventions including gestation and/or with more obesogenic post-weaning diets (e.g., high fat/high sucrose diet).

Our main finding shows that a suboptimal ALA intake during suckling/weaning promotes the susceptibility to an increased adiposity later in life. It is important to note that the ALA diet (0.8% total FA; 0.16 g ALA/100 g diet), used for the low intake group, does not correspond to a deficient diet (0.01 g/100 g diet) as previously described [41]. Interestingly, smoking mothers have lower ALA content in breast milk than non-smoking ones [42] and maternal smoking is associated with an increased risk of late onset overweight [43]. Consequently, if relevant to the human newborn, our findings suggest the need of recommending n-3 PUFA supplementation in the pregnant/lactating mother with a low ALA intake or at risk of suboptimal breast milk content.

## Competing interests

EP, OA, ECG, JM, GP and KM authors work for Nestle Company.

## Authors' contributions

EP wrote the manuscript and designed the use of the deuterium approach. OA designed and conducted the study. CG and CC developed the specific insulin analysis for GP. DR and CPA contributed with the eicosanoid analyses. ECG interpreted the data and drafted the manuscript. JM and GP performed the statistics. CB and ST developed the deuterium approach and did the analyses. KM directed the study and wrote the manuscript. All authors read and approved the final manuscript.

## Supplementary Material

Additional file 1 **Table S1 on "Composition of milk formula and weaning diets"**. The file contains one table. Click here for file

Additional file 2 **Table S2 on "Fatty acid composition of the suckling/weaning diets (milk formula and pellets)"**. The file contains one table. Click here for file

Additional file 3 **Table S3 on "Adipose tissue (AT) weight (% body weight) at d21 and d136"**. The file contains one table. Click here for file

Additional file 4 **Table S4 on "Fractional proliferation rate of cells (in % new cells/5 days) in adipose tissue (AT) at d21 and d136"**. The file contains one table. Click here for file

Additional file 5 **Table S5 on "De novo lipogenesis (DNL) and fractional synthesis rate (FSR) of TG in the liver and adipose tissues (AT) at d21 and d136"**. The file contains one table. Click here for file

Additional file 6 **Table S6 on "Eicosanoid and AA concentrations (ng/50 mg tissue) in retroperitoneal and subcutaneous AT at d21 and d136"**. The file contains one table. Click here for file
