# Supplementary material for: A low α-linolenic intake during early life increases adiposity in the adult guinea pig
Source: Nutr Metab (Lond). 2010 Jan 29;7:8. doi: 10.1186/1743-7075-7-8 (PMC2825514; doi:10.1186/1743-7075-7-8)
Supplement: Additional file 1 — Table S1 on "Composition of milk formula and weaning diets". The file contains one table. [file 1743-7075-7-8-S1.DOC]

**Table S1: Composition of milk formula and weaning diets**

| Ingredient | Milk formula ^1^ | | |  | Pellet ^1^ | | |
| --- | --- | --- | --- | --- | --- | --- | --- |
|  | g/100 ml | | |  | g/100g | | |
|  | 10%-ALA | 2.4%-ALA | 0.8%-ALA |  | 10%-ALA | 2.4%-ALA | 0.8%-ALA |
| Whey^2^ | 1.04 | 1.04 | 1.04 |  | 7.5 | 7.5 | 7.5 |
| Calcium caseinate^3^ | 4.64 | 4.64 | 4.64 |  | 33.7 | 33.7 | 33.7 |
| DL-Methionine^4^ | 0.08 | 0.08 | 0.08 |  | 0.6 | 0.6 | 0.6 |
| Lactose^4^ | 3.36 | 3.36 | 3.36 |  | 24.4 | 24.4 | 24.4 |
| Vitamin Mix (350001)^5^ | 0.16 | 0.16 | 0.16 |  | 1.2 | 1.2 | 1.2 |
| Mineral Mix (250001)^5^ | 1.20 | 1.20 | 1.20 |  | 8.7 | 8.7 | 8.7 |
| Vitamin C^4^ | 0.06 | 0.06 | 0.06 |  | 0.5 | 0.5 | 0.5 |
| Choline chloride^4^ | 0.03 | 0.03 | 0.03 |  | 0.2 | 0.2 | 0.2 |
| Coconut oil^6^ | 0.41 | 0.47 | 0.45 |  | 3.0 | 3.4 | 3.3 |
| Corn oil^6^ | 0.61 | 1.15 | 1.33 |  | 4.4 | 8.4 | 9.6 |
| Palm oil^6^ | 1.40 | 1.30 | 1.27 |  | 10.2 | 9.5 | 9.2 |
| Rapeseed oil^6^ | 0.23 | 0.17 | 0.15 |  | 1.7 | 1.3 | 1.1 |
| Linseed oil^6^ | 0.55 | 0.10 | - |  | 4.0 | 0.7 | - |
| Water^7^ | 86 | 86 | 86 |  | - | - | - |

^1^Treatments were isocaloric .

^2^Harlan Teklad (Basel, Switzerland); ^3^Lacprodan, Arla Foods (Hellerup, Denmark), ^4^Nestec SA (Konolfingen, Switzerland) ; ^5^Dyets Inc., (Bethlehem, PA, USA); ^6^Sofinol S.A. (Manno, Switzerland); ^7^Vittel (Vittel, France).
