# Supplementary material for: A low α-linolenic intake during early life increases adiposity in the adult guinea pig
Source: Nutr Metab (Lond). 2010 Jan 29;7:8. doi: 10.1186/1743-7075-7-8 (PMC2825514; doi:10.1186/1743-7075-7-8)
Supplement: Additional file 2 — Table S2 on "Fatty acid composition of the suckling/weaning diets (milk formula and pellets)". The file contains one table. [file 1743-7075-7-8-S2.DOC]

**Table S2: Fatty acid composition of the suckling/weaning diets (milk formula and pellets)**

|  | 10%-ALA | 2.4%-ALA | 0.8%-ALA |
| --- | --- | --- | --- |
|  | % of total fatty acids | | |
| C12:0 | 7.1 | 8.2 | 7.9 |
| C14:0 | 3.4 | 3.9 | 3.7 |
| C16:0 | 22.7 | 22.0 | 21.8 |
| C16:1 | 0.2 | 0.1 | 0.1 |
| C18:0 | 3.0 | 3.2 | 3.0 |
| C18:1 | 33.5 | 33.0 | 33.3 |
| C18:2n-6 (LA) | 20.2 | 26.2 | 28.5 |
| C18:3n-3 (ALA) | 9.9 | 2.4 | 0.8 |
| C20:0 | 0.0 | 0.0 | 0.0 |
| C20:1 | 0.0 | 0.0 | 0.0 |
| C20:4n-6 | 0.0 | 0.0 | 0.0 |
| C20:5n-3 | 0.0 | 0.0 | 0.0 |
| C22:0 | 0.0 | 0.0 | 0.0 |
| C22:5n-3 | 0.0 | 0.0 | 0.0 |
| C22:6n-3 | 0.0 | 0.0 | 0.0 |
| LA :ALA | 2.0 | 10.9 | 35.6 |
