# Supplementary material for: A low α-linolenic intake during early life increases adiposity in the adult guinea pig
Source: Nutr Metab (Lond). 2010 Jan 29;7:8. doi: 10.1186/1743-7075-7-8 (PMC2825514; doi:10.1186/1743-7075-7-8)
Supplement: Additional file 3 — Table S3 on "Adipose tissue (AT) weight (% body weight) at d21 and d136". The file contains one table. [file 1743-7075-7-8-S3.DOC]

**Table S3: Adipose tissue (AT) weight (% body weight) at d21 and d136**

|  | Interscapular AT | | Epididymal AT | | Retroperitoneal AT | |
| --- | --- | --- | --- | --- | --- | --- |
| Groups | d21 | d136 | d21 | d136 | d21 | d136 |
| 10%-ALA | 0.426 ± 0.110 | 1.63 ± 0.07^ab^ | 0.052 ± 0.011 | 0.96 ± 0.09^a^ | 0.102 ± 0.021 | 1.08 ± 0.08 |
| 2.4%-ALA | 0.618 ± 0.090 | 1.54 ± 0.06^a^ | 0.059 ± 0.006 | 0.99 ± 0.08^ab^ | 0.120 ± 0.028 | 1.12 ± 0.07 |
| 0.8%-ALA | 0.480 ± 0.037 | 1.72 ± 0.07^b^ | 0.049 ± 0.010 | 1.15 ± 0.07^b^ | 0.118 ± 0.020 | 1.19 ± 0.03 |

Data are medians ± SEmedian, n=10 /group. At d21 and d136 n=5 and 15/group, respectively. Different superscript letters indicate treatment differences at p < 0.05.
