# Supplementary material for: A low α-linolenic intake during early life increases adiposity in the adult guinea pig
Source: Nutr Metab (Lond). 2010 Jan 29;7:8. doi: 10.1186/1743-7075-7-8 (PMC2825514; doi:10.1186/1743-7075-7-8)
Supplement: Additional file 4 — Table S4 on "Fractional proliferation rate of cells (in % new cells/5 days) in adipose tissue (AT) at d21 and d136". The file contains one table. [file 1743-7075-7-8-S4.DOC]

**Table S4: Fractional proliferation rate of cells (in % new cells / 5 days) in adipose tissue (AT) at d21 and d136**

|  | Subcutaneous AT | | Epididymal AT | | Retroperitoneal AT | |
| --- | --- | --- | --- | --- | --- | --- |
| Groups | d21^*^ | d136 | d21 | d136 | d21 | d136 |
| 10%-ALA | 8.9 ± 8.7 | 8.6 ± 1.7^a^ | 13.5 ± 2.0 | 7.7 ± 1.3 | 5.3 ± 2.3 | 4.0 ± 0.6 |
| 0.8%-ALA | 18.7 ± 13.7 | 15.2 ± 1.3^b^ | 15.0 ± 4.1 | 7.8 ± 2.6 | 3.3 ± 0.7 | 7.9 ± 2.3 |

Data are medians ± SEmedian, n=10 /group except * n = 3 for 10%-ALA and n = 4 for 0.8%-ALA group, respectively. Different superscript letters indicate treatment differences at p < 0.05.
