# Supplementary material for: A low α-linolenic intake during early life increases adiposity in the adult guinea pig
Source: Nutr Metab (Lond). 2010 Jan 29;7:8. doi: 10.1186/1743-7075-7-8 (PMC2825514; doi:10.1186/1743-7075-7-8)
Supplement: Additional file 5 — Table S5 on "De novo lipogenesis (DNL) and fractional synthesis rate (FSR) of TG in the liver and adipose tissues (AT) at d21 and d136". The file contains one table. [file 1743-7075-7-8-S5.DOC]

**Table S5: De novo lipogenesis (DNL) and fractional synthesis rate (FSR) of TG in the liver and adipose tissues (AT) at d21 and d136**

|  | Liver | | Subcutaneous AT | | Epididymal AT | | Retroperitoneal AT | | |
| --- | --- | --- | --- | --- | --- | --- | --- | --- | --- |
| Groups | d21 | d136 | d21^*^ | d136 | d21 | d136 | d21 | d136 | |
| DNL (% new palmitate / 5 days) | | | | | | | | | |
| 10%-ALA | 8.8 ± 2.6^a^ | 5.7 ± 0.6 | 9.3 ± 6.6 | 1.87 ± 0.26 | 14.9 ± 2.8 | 1.18 ± 0.16 | 13.8 ± 5.4 | 1.40 ± 0.39 | |
| 0.8%-ALA | 17.4 ± 3.7^b^ | 5.6 ± 0.4 | 2.2 ± 1.8 | 1.29 ± 0.18 | 17.1 ± 2.5 | 0.92 ± 0.12 | 12.9 ± 2.3 | 1.06 ± 0.07 | |
| FSR of TG (% new TG / 5 days) | | | | | | | | |  |
| 10%-ALA | 65.6 ± 5.1 | 25.9 ± 2.4 | 13.9 ± 5.4 | 7.2 ± 0.5 | 19.0 ± 1.2 | 4.6 ± 0.5 | 27.7 ± 2.5 | 6.7 ± 0.3 | |
| 0.8%-ALA | 62.7 ± 5.3 | 27.2 ± 2.2 | 12.4 ± 4.6 | 6.9 ± 0.5 | 22.4 ± 2.1 | 4.7 ± 0.2 | 25.8 ± 3.7 | 6.6 ± 0.7 | |

Data are medians ± SEmedian. n=10 /group except * n = 3 for 10%-ALA and n = 4 for 0.8%-ALA group. Different superscript letters indicate statistical significance at p < 0.05.
