# Supplementary material for: A low α-linolenic intake during early life increases adiposity in the adult guinea pig
Source: Nutr Metab (Lond). 2010 Jan 29;7:8. doi: 10.1186/1743-7075-7-8 (PMC2825514; doi:10.1186/1743-7075-7-8)
Supplement: Additional file 6 — Table S6 on "Eicosanoid and AA concentrations (ng/50 mg tissue) in retroperitoneal and subcutaneous AT at d21 and d136". The file contains one table. [file 1743-7075-7-8-S6.DOC]

**Table S6: Eicosanoid and AA concentrations (ng/50 mg tissue) in retroperitoneal and subcutaneous AT at d21 and d136**

|  | d21 | | |  | d136 | | |
| --- | --- | --- | --- | --- | --- | --- | --- |
|  | 10%-ALA | 2.4%-ALA | 0.8%-ALA |  | 10%-ALA | 2.4%-ALA | 0.8%-ALA |
|  | *Retroperitoneal AT* | | | | | | |
| 6 Keto-PGF_1α_ | 1.83 ± 0.36 | 1.95 ± 0.02 | 2.05 ± 0.15 |  | 0.34 ± 0.07 | 0.18 ± 0.04 | 0.20 ± 0.02 |
| PGF_2α_ | 0.15 ± 0.04 | 0.21 ± 0.13 | 0.13 ± 0.02 |  | 0.04 ± 0.01 | 0.04 ± 0.01 | 0.04 ± 0.01 |
| PGE_2_ | 0.44 ± 0.09 | 0.83 ± 0.35 | 0.59 ± 0.13 |  | 0.87 ± 0.75^ab^ | 1.78 ± 0.03^a^ | 0.12 ± 0.05^b^ |
| PGD_2_ | 0.24 ± 0.04 | 0.54 ± 0.27 | 0.35 ± 0.06 |  | 0.09 ± 0.02 | 0.10 ± 0.01 | 0.07 ± 0.01 |
| PGJ_2_ | 0.01 ± 0.01 | 0.02 ± 0.01 | 0.01 ± 0.01 |  | 1.07 ± 1.01^a^ | 2.51 ± 0.42^b^ | 0.01 ± 0.01^a^ |
| TXB_2_ | 1.16 ± 0.38 | 0.44 ± 0.03 | 0.89 ± 0.17 |  | 0.12 ± 0.02 | 0.11 ± 0.03 | 0.12 ± 0.02 |
| LTB_4_ | 0.08 ± 0.02 | 0.08 ± 0.01 | 0.07 ± 0.02 |  | 0.04 ± 0.01 | 0.04 ± 0.01 | 0.05 ± 0.01 |
| AA | 206.0 ± 34.5 | 206.0 ± 18.5 | 256.0 ± 17.0 |  | 49.7 ± 5.5 | 48.9 ± 8.5 | 42.3 ± 4.4 |
|  | *Subcutaneous AT* | | | | | | |
| 6 Keto-PGF_1α_ | 0.39 ± 0.12 | 0.50 ± 0.13 | 0.65 ± 0.08 |  | 0.22 ± 0.06 | 0.14 ± 0.03 | 0.14 ± 0.04 |
| PGF_2α_ | 0.07 ± 0.01 | 0.11 ± 0.03 | 0.12 ± 0.01 |  | 0.03 ± 0.01 | 0.02 ± 0.01 | 0.02 ± 0.01 |
| PGE_2_ | 0.35 ± 0.08 | 0.44 ± 0.12 | 0.55 ± 0.14 |  | 0.15 ± 0.06 | 0.06 ± 0.02 | 0.09 ± 0.03 |
| PGD_2_ | 0.40 ± 0.14 | 0.40 ± 0.07 | 0.50 ± 0.03 |  | 0.11 ± 0.04 | 0.06 ± 0.01 | 0.07 ± 0.01 |
| PGJ_2_ | 0.01 ± 0.01 | 0.01 ± 0.01 | 0.01 ± 0.01 |  | 0.01 ± 0.01 | 0.01 ± 0.01 | 0.01 ± 0.01 |
| TXB_2_ | 0.45 ± 0.14 | 0.49 ± 0.13 | 0.78 ± 0.02 |  | 0.22 ± 0.07 | 0.12 ± 0.02 | 0.17 ± 0.05 |
| LTB_4_ | 0.07 ± 0.05 | 0.06 ± 0.04 | 0.06 ± 0.04 |  | 0.01 ± 0.01 | 0.01 ± 0.01 | 0.02 ± 0.02 |
| AA | 149.6 ± 27.1 | 134.4 ± 14.0 | 162.6 ± 26.3 |  | 45.9 ± 10.6 | 39.1 ± 4.0 | 51.5 ± 9.4 |

Data are medians ± SEmedian, n = 5 and 10/group at d21 and d136, respectively, except for retroperitoneal AT n = 3 at d21. Different superscript letters indicate statistical significance at p < 0.05.
